# Predictive biomarkers for the efficacy of peptide vaccine treatment: based on the results of a phase II study on advanced pancreatic cancer

**DOI:** 10.1186/s13046-017-0509-1

**Published:** 2017-02-28

**Authors:** Yoshitaro Shindo, Shoichi Hazama, Nobuaki Suzuki, Haruo Iguchi, Kazuhiro Uesugi, Hiroaki Tanaka, Atsushi Aruga, Takashi Hatori, Hidenobu Ishizaki, Yuzo Umeda, Toshiyoshi Fujiwara, Tetsuya Ikemoto, Mitsuo Shimada, Kazuhiko Yoshimatsu, Hiroko Takenouchi, Hiroto Matsui, Shinsuke Kanekiyo, Michihisa Iida, Yasunobu Koki, Hideki Arima, Hiroyuki Furukawa, Tomio Ueno, Shigefumi Yoshino, Tomonobu Fujita, Yutaka Kawakami, Yusuke Nakamura, Masaaki Oka, Hiroaki Nagano

**Affiliations:** 1 Department of Gastroenterological, Breast and Endocrine Surgery, Yamaguchi University Graduate School of Medicine, 1-1-1 Minami-Kogushi, Ube, Yamaguchi 755-8505 Japan; 2 Department of Translational Research and Developmental Therapeutics against Cancer, Yamaguchi University School of Medicine, Ube, Japan; 3 Clinical Research Center, Shikoku Cancer Center, NHO., Matsuyama, Japan; 4 Department of Surgical Oncology, Osaka City University Graduate School of Medicine, Osaka, Japan; 5 Institute of Gastroenterology, Tokyo Women’s Medical University, Tokyo, Japan; 6 Department of Surgical Oncology and Regulation of Organ Function, Miyazaki University School of Medicine, Miyazaki, Japan; 7 Department of Gastroenterological Surgery, Okayama University Graduate School of Medicine, Okayama, Japan; 8 Department of Digestive and Transplant Surgery, Tokushima University Graduate School of Medicine, Tokushima, Japan; 9 Department of Surgery, Tokyo Women’s Medical University Medical Center East, Tokyo, Japan; 10 Department of Pharmacy, Yamaguchi University Hospital, Ube, Japan; 11 Division of Cellular Signaling, Institute for Advanced Medical Research, Keio University School of Medicine, Tokyo, Japan; 12 Section of Hematology/Oncology, Department of Medicine, The University of Chicago, Chicago, Illinois USA; 13 Yamaguchi University, Yamaguchi, Japan

**Keywords:** Pancreatic cancer, Peptide vaccine, Predictive biomarker, PD-1, Tim-3

## Abstract

**Background:**

The purpose of the present study was to explore novel biomarkers that can predict the clinical outcome of patients before treatment or during vaccination. These would be useful for the selection of appropriate patients who would be expected to exhibit better treatment outcomes from vaccination, and for facilitating the development of cancer vaccine treatments.

**Methods:**

From a single-arm, non-randomized, human leukocyte antigen (HLA)-A-status-blind phase II trial of a vaccine treatment using three HLA-A*2402-restricted peptides for advanced pancreatic cancer (PC), we obtained peripheral blood samples from 36 patients of an HLA-A*2402-matched group and 27 patients of an HLA-A*2402-unmatched group.

**Results:**

Multivariate analysis (HR = 2.546; 95% CI = 1.138 to 5.765; *p* = 0.0231) and log-rank test (*p* = 0.0036) showed that a high expression level of programmed death-1 (PD-1) on CD4+ T cells was a negative predictive biomarker of overall survival in the HLA-A*2402-matched group . Moreover, a high expression level of PD-1 on CD4+ T cells was a negative predictor for the induction of cytotoxic T lymphocytes (*p* = 0.0007). After treatment, we found that the upregulation of PD-1 and T cell immunoglobulin mucin-3 (Tim-3) expression on CD4+ and CD8+ T cells was significantly associated with a poor clinical outcome in the HLA-A*2402-matched group (*p* = 0.0330, 0.0282, 0.0046, and 0.0068, respectively). In contrast, there was no significant difference for these factors in the HLA-A*2402-unmatched group.

**Conclusions:**

Our results indicate that the upregulation of PD-1 and Tim-3 expression on CD4+ and CD8+ T cells may restrict T cell responses in advanced PC patients; therefore, combination immunotherapy with blockade of PD-1 and Tim-3 to restore T cell responses may be a potential therapeutic approach for advanced PC patients.

**Trial registration:**

Clinical-Trail-Registration: UMIN000008082.

**Electronic supplementary material:**

The online version of this article (doi:10.1186/s13046-017-0509-1) contains supplementary material, which is available to authorized users.

## Background

Pancreatic cancer (PC) is one of the most lethal cancers, and the majority of PC patients are diagnosed at an advanced stage due to the difficulty of early diagnosis [1]. It has been reported that advanced PC patients have a median survival time (MST) of less than 6 months [2]. Gemcitabine (GEM) has been regarded as a standard chemotherapeutic agent for advanced PC [3]. Although recent advances in combination chemotherapy including GEM and other cytotoxic agents or chemoradiotherapy have improved the clinical outcomes of advanced PC patients, the prognosis still remains poor [4–7]. Therefore, new treatment strategies are necessary.

Recent advances in cancer immunotherapies, such as immune checkpoint inhibitors, have shown some durable clinical responses in patients with various types of advanced cancers [8, 9]. However, since their clinical efficacy remains limited, active immunotherapies using tumor-associated antigen (TAA)-derived epitope peptides, which can induce tumor-specific cytotoxic T lymphocytes (CTLs) in vivo, should be developed. The efficacy of current immunotherapies also remains limited due to the immunosuppressive tumor microenvironment, which leads to TAA-specific T cell exhaustion or anergy and the escape of tumor cells from immune attack [10]. It has been reported that the expression of programmed death-1 (PD-1) and T cell immunoglobulin mucin-3 (Tim-3), which are inhibitory receptors, is upregulated on exhausted T cells in cancer patients [11, 12]. Regulatory T cells (Tregs) and myeloid-derived suppressor cells (MDSCs) are considered to be pivotal components of immunosuppressive cells [13, 14]. Hence, there is a desperate need to identify predictive biomarkers that can enable, prior to treatment, the selection of patients who are likely to respond well and effectively to epitope peptides that induce specific CTLs [15–19].

We have reported a phase II study (VENUS-PC study) in which three epitope peptides (one derived from oncoantigen KIF20A (RAB6KIFL)[20] and two derived from vascular endothelial growth factor receptors (VEGFRs)[21, 22]) in combination with GEM were applied to advanced PC patients [23]. We verified the safety of the treatment and its potential to induce CTLs. We also revealed that a high CTL response after vaccination and an injection site skin reaction were possible biomarkers for a long survival in vaccinated patients [23].

The purpose of the present study was to explore novel biomarkers for predicting the efficacy of immunotherapies, and to apply such information to select patients who are expected to exhibit better treatment outcomes following vaccination. Here, we report the results of possible biomarkers for active immunotherapies and the need for overcoming immune suppression.

## Methods

### Patients and study design

**Fig. 1:**
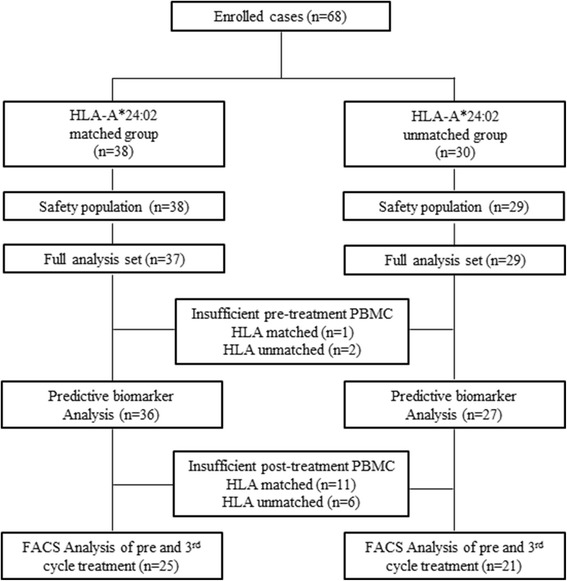
CONSORT diagram. Scheme showing an HLA-A-status double-blind, biologically randomized phase II study of three therapeutic epitope peptides combined with gemcitabine as a first-line therapy for advanced pancreatic cancer (VENUS-PC study)

The detailed protocol of this phase II study has been reported recently (VENUS-PC study) [23]. Briefly, the therapy consisted of a cocktail of three therapeutic epitope peptides in addition to GEM. Although the peptides used in this study were human leukocyte antigen (HLA)-A*2402-restricted peptides, all enrolled patients, whose HLA-A status was double-blinded, were administrated the same regime of peptide cocktail and GEM. Each of the three peptides derived from KIF20A-66 (KVYLRVRPLL)[20] (3 mg/shot), VEGFR1-1084 (SYGVLLWEI)[24] (2 mg/shot), and VEGFR2-169 (RFVPDGNRI)[25] (2 mg/shot) was mixed with 1.0 ml of incomplete Freund’s adjuvant (Montanide ISA51; Seppic, Paris, France) and administered subcutaneously into the thigh or axilla region once a week for the first 8 weeks, and then once every 2 weeks. GEM was administered at a dose of 1000 mg/m^2^ on days 1, 8, and 15 in a 28-day cycle. The patients were eligible for enrollment if they were 20 years of age or older with a histologically or cytologically confirmed advanced PC, were naïve for chemotherapy, had adequate functions of critical organs, and had a life expectancy of 3 months or more. Written informed consent was obtained from each patient at the time of enrollment. The study was carried out in accordance with the Declaration of Helsinki on experimentations involving human subjects, was approved by the Institutional Ethics Review Boards of Yamaguchi University (H24-14) at each study site, and was registered in the UMIN Clinical Trials Registry as UMIN000008082. Among the 68 patients who were enrolled in this study, 63 patients, for whom peripheral blood mononuclear cell (PBMC) samples were sufficiently stocked, were evaluated in this study, and 46 patients, for whom sufficient post-treatment PBMC samples were available, were analyzed (Fig. 1).

### Sample collection

A complete blood count and serum chemistry tests were performed before treatment and every 2 weeks. For PBMC and blood plasma isolation, 35 ml of blood was drawn before each course. PBMCs were enriched by density gradient centrifugation with Ficoll-Paque (Amersham Pharmacia Biotech, Uppsala, Sweden). The PBMCs and plasma were preserved in a liquid nitrogen tank until examination.

### Flow cytometry

After washing the PBMCs in FACS buffer (phosphate-buffered saline, 1% fetal bovine serum, and 0.5 mmol/l ethylenediaminetetraacetic acid), the following antibodies were used for flow cytometry: VioBlue-conjugated anti-human CD4 (clone VIT4; Miltenyi Biotec, Bergisch Gladbach, Germany), FITC-conjugated anti-human CD8 (clone RPA-T8; BD Biosciences, Heidelberg, Germany) and CD25 (clone B1.49.9; Beckman Coulter, Marseille, France), APC-conjugated anti-human PD-1 (clone EH12.2H7; Biolegend, San Diego, CA) and CD45RA (clone HI100; Biolegend), and PE-conjugated anti-human Tim-3 (clone F38-2E2; Biolegend). After staining, the cells were washed in FACS buffer and analyzed using a MACSQuant flow cytometer with MACSQuantify software (Miltenyi Biotec). In this study, the percentages of PD-1+ and Tim-3+ T cells were calculated as percentages of the total CD4+ or CD8+ T cells. Tregs were identified as CD4+ CD45RA- CD25high cells [26] and were calculated as a percentage of the CD4+ lymphocytes. MDSCs were identified as CD11b + CD33+ cells [27] and were calculated as a percentage of the total PBMCs.

### Measurement of the peptide-specific interferon-γ response and plasma interleukin-6 (IL-6) level

Antigen-specific T cell responses were estimated by enzyme-linked immunospot assays following in vitro sensitization [28]. The numbers of peptide-specific spots were calculated by subtracting the spot number in the control well from the spot number of wells with vaccinated peptide-pulsed stimulator cells. Antigen-specific T cell responses were classified into four grades (-, +, ++, or +++) according to the algorithm flow chart described in our previous report (Additional file 1: Figure S1) [29, 30]. Plasma IL-6 levels were measured by electrochemiluminescence immunoassays (Meso Scale Discovery, Rockville, MD) according to the manufacturer’s instructions.

### Statistical analysis

Results are expressed as the means ± standard error. Categorical variables were compared by using Chi-square and Fisher’s exact tests. Survival curves were analyzed by the Kaplan-Meier method and the log-rank test. Potential prognostic factors for survival were determined by univariate analysis, and were assessed by multivariate analysis with the Cox proportional hazards model. The Wilcoxon matched-pairs test, Mann-Whitney U-tests, and Spearman test were used to assess the differences and correlation were used to assess the differences between the study groups. Statistical analyses were performed with JMP V11 (SAS, Cary, NC) and GraphPad Prism V5.0 (GraphPad Software, Inc., San Diego, CA). A *p* < 0.05 was considered to be statistically significant.

## Results

### Clinical outcomes

Sixty-three patients who had a sufficient PBMC sample were evaluated in this study (Fig. 1). The patient characteristics are summarized in Table 1. There were no significant differences between the patients of the HLA-A*2402-matched group and the patients of the HLA-A*2402-unmatched group for age, gender, disease stage, and tumor markers (Table 1).

**Table 1:** Baseline characteristics

	HLA-A*24:02	HLA-A*24:02	*p*-value
	matched	unmatched	
Number of patients	36	27	
Age, years	62.9 ± 2.1	63.4 ± 2.1	0.7072
Gender			0.3074
Male	17 (47.2%)	17 (63.0%)	
Female	19 (52.8%)	10 (37.0%)	
Stage (UICC)			0.7439
III	7 (19.4%)	5 (18.5%)	
IV	26 (72.2%)	21 (77.8%)	
Recurrence	3 (8.3%)	1 (3.7%)	
Tumor marker			
CEA	369.2 ± 247.9	8.5 ± 2.1	0.1722
CA19-9	3870.1 ± 1972.7	3643.2 ± 1444.7	0.0718

Abbreviations: *HLA* human leukocyte antigen, *UICC* Union for International Cancer Control, *CEA* carcinoembryonic antigen, *CA19-9* carbohydrate antigen 19-9

### Predictive factors affecting overall survival (OS) with immunotherapy in the HLA-A*2402-matched group

**Fig. 2:**
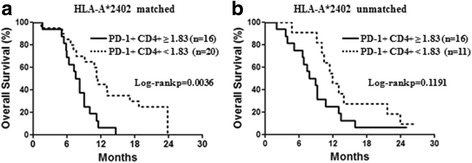
Overall survival according to a biomarker. Overall survival rates of patients in the HLA-A*2402-matched group (**a**) and HLA-A*2402-unmatched group (**b**) were analyzed by the Kaplan-Meier method for low (*dotted line*) or high (*solid line*) expression levels of PD-1 on CD4+ T cells

We classified the patients into two groups: a long-survival group (patients with a survival of >1 year) and a short-survival group (patients with a survival of <1 year). To explore predictive biomarkers for this vaccine therapy, we analyzed the parameters of age, gender, disease stage, hemoglobin (Hb), neutrophil-lymphocyte ratio (NLR), carcinoembryonic antigen (CEA), carbohydrate antigen 19-9 (CA19-9), IL-6, PD-1+ CD4+ T cells, Tim-3+ CD4+ T cells, PD-1+ CD8+ T cells, Tim-3+ CD8+ T cells, Tregs, and MDSCs in the HLA-A*2402-matched group. The applied cutoffs for the assessed parameters were derived based on the median values. In the univariate analysis, age (≥65 years; hazard ratio (HR) = 2.150; 95% confidence interval (CI) = 1.058 to 4.396; *p* = 0.0345) and the expression level of PD-1 on CD4+ T cells (≥1.83; HR = 2.962; 95% CI = 1.383 to 6.471; *p* = 0.0054) were significant prognostic factors associated with OS (Table 2). In the multivariate analysis with the Cox proportional hazards model, only the expression level of PD-1 on CD4+ T cells (≥1.83; HR = 2.546; 95% CI = 1.138 to 5.765; *p* = 0.0231) remained associated with poor OS (Table 2). In the HLA-A*2402-matched group, the 1-year survival rate and MST of the 16 patients with a high expression level of PD-1 on CD4+ T cells were significantly worse than those of the 20 patients with a low expression level of PD-1 on CD4+ T cells (6.3% vs. 45.0% and 7.9 months vs. 11.3 months, respectively; log-rank test, *p* = 0.0036; Fig. 2a). In contrast, among the 27 patients of the HLA-A*2402-unmatched group, there was no difference in these parameters between those with a high or low expression level of PD-1 on CD4+ T cells (Fig. 2b; *p* = 0.1191).

**Table 2:** Univariate and multivariate analyses of overall survival (*n* = 36 HLA-A*2402-matched patients)

Variables		Univariate analysis			Multivariate analysis		
		Hazard ratio	95% CI	*p*-value	Hazard ratio	95% CI	*p*-value
Age	≥65	2.150	1.058 to 4.396	**0.0345**	1.691	0.798 to 3.588	0.1689
Gender	male/female	1.193	0.581 to 2.436	0.6271			
Stage	III/IV and Recurrence	0.743	0.275 to 1704	0.5033			
Hb	≥13.2	0.724	0.350 to 1.515	0.3859			
NLR	≥2.48	1.514	0.756 to 3.018	0.2384			
CEA	≥5.3	1.791	0.879 to 3.813	0.1095			
CA19-9	≥541	1.853	0.862 to 3.935	0.1120			
IL-6	≥0.97	0.906	0.454 to 1.851	0.7816			
PD-1+ CD4+	≥1.83	2.962	1.383 to 6.471	**0.0054**	2.546	1.138 to 5.765	**0.0231**
Tim-3+ CD4+	≥2.54	0.741	0.362 to 1.522	0.4091			
PD-1+ CD8+	≥4.73	1.892	0.925 to 3.932	0.0803			
Tim-3+ CD8+	≥4.58	0.881	0.429 to 1.799	0.7269			
Treg	≥1.93	0.880	0.420 to 1.794	0.7268			
MDSC	≥15.07	1.267	0.638 to 2.555	0.4981			

Statistical significant results are highlighted in bold letters

Abbreviations: *HLA* human leukocyte antigen, *CI* confidence interval, *Hb* hemoglobin, *CEA* carcinoembryonic antigen, *CA19-9* carbohydrate antigen 19-9, *NLR* neutrophil lymphocyte ration, *IL-6* interleukin-6, *PD-1* Programmed death-1, *Tim-3* T cell immunoglobulin mucin-3, *Treg* Regulatory T cell, *MDSC* Myeloid-derived suppressor cell

### Relationship to CTL induction

To compare the prognostic factors according to the numbers of peptide-specific response, the parameters of age, gender, disease stage, NLR, C-reactive protein (CRP), IL-6, PD-1+ CD4+ T cells, Tim-3+ CD4+ T cells, PD-1+ CD8+ T cells, Tim-3+ CD8+ T cells, Tregs, and MDSCs were evaluated in the HLA-A*2402-matched group. We conducted a vaccine trial using multiple epitope peptides. Therefore, we compared these factors according to multiple CTL responses, because it has been reported that high CTL responses to multiple peptides are associated with better prognosis [29, 31]. A significantly high NLR (3.61 ± 0.32 vs. 2.14 ± 0.16; *p* = 0.0007), high expression level of PD-1 on CD4+ T cells (3.46 ± 0.56 vs. 1.58 ± 0.17; *p* = 0.0007), and high number of Tregs (2.41 ± 0.28 vs. 1.64 ± 0.13; *p* = 0.0121) were observed in the low-CTL-response group when compared to the high-CTL-response group (Table 3).

**Table 3:** Comparison of prognostic factors according to the numbers of peptide-specific responses (*n* = 36 HLA-A*2402-matched patients)

Variables	The number of peptide specific response		*p*-value
	0 or 1	2 or 3	
the number of patients	10	26	
Age	69.3 ± 3.7	60.5 ± 2.4	0.1075
Gender			0.4629
Male	6	11	
Female	4	15	
Stage (UICC)			0.5209
III	2	5	
IV	8	18	
Recurrence	0	3	
NLR	3.61 ± 0.32	2.14 ± 0.16	**0.0007**
CRP	1.39 ± 0.48	0.58 ± 0.17	0.1425
IL-6	2.11 ± 0.70	19.40 ± 17.32	0.7640
PD-1+ CD4+ T cell	3.46 ± 0.56	1.58 ± 0.17	**0.0007**
Tim-3+ CD4+ T cell	3.30 ± 0.53	3.71 ± 0.68	0.8184
PD-1+ CD8+ T cell	5.63 ± 0.74	4.05 ± 0.45	0.0689
Tim-3+ CD8+ T cell	5.37 ± 0.98	4.77 ± 0.49	0.7108
Treg	2.41 ± 0.28	1.64 ± 0.13	**0.0121**
MDSC	17.34 ± 1.70	14.29 ± 0.78	0.1005

Statistical significant results are highlighted in bold letters

Abbreviations: *HLA* human leukocyte antigen, *CI* confidence interval, *UICC* Union for International Cancer, *NLR* neutrophil lymphocyte ration, *CRP* C-reactive protein, *IL-6* interleukin-6, *PD-1* Programmed death-1, *Tim-3* T cell immunoglobulin mucin-3, *Treg* Regulatory T cell, *MDSC* Myeloid-derived suppressor cell

Next, we evaluated these factors according to the patients who showed no CTL response and the patients who showed CTL responses to one or more peptides. A significantly high NLR (3.91 ± 0.49 vs. 2.33 ± 0.17; *p* = 0.0153), and CRP (1.89 ± 0.63 vs. 0.63 ± 0.18; *p* = 0.0153) were observed in the low-CTL-response group when compared to the high-CTL-response group (Additional file 2: Table S1).

### PD-1 and Tim-3 expression on CD4+ and CD8+ T cells after the 3rd cycle of treatment

**Fig. 3:**
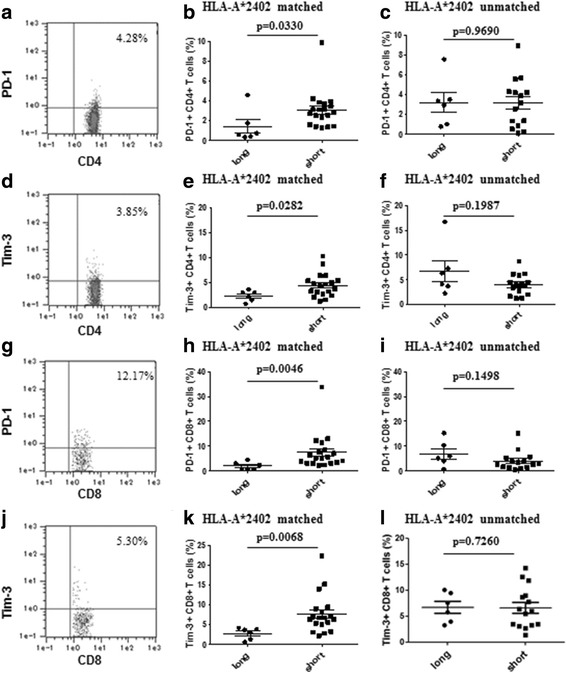
Expression of biomarkers after 3rd cycle treatment. PD-1 and Tim-3 expression on CD4+ and CD8+ T cells obtained from patients in the HLA-A*2402-matched group and the HLA-A*2402-unmatched group after the 3rd cycle of treatment. (**a**) Analysis for PD-1 expression in the CD4+ lymphocyte gate. (**b**) In the HLA-A*2402-matched group, the percentage of PD-1+ CD4+ T cells in the patients of the short-survival group (*n* = 19) was significantly higher than in the patients of the long-survival group (*n* = 6). (**d**) Analysis for Tim-3 expression in the CD4+ lymphocyte gate. (**e**) In the HLA-A*2402-matched group, the percentage of Tim-3+ CD4+ T cells in the patients of the short-survival group was significantly higher than in the patients of the long-survival group. (**g**) Analysis for PD-1 expression in the CD8+ lymphocyte gate. (**h**) In the HLA-A*2402-matched group, the percentage of PD-1+ CD8+ T cells in the patients of the short-survival group was significantly higher than in the patients of the long-survival group. (**j**) Analysis for Tim-3 expression in the CD8+ lymphocyte gate. (**k**) In the HLA-A*2402-matched group, the percentage of Tim-3+ CD8+ T cells in the patients of the short-survival group was significantly higher than in the patients of the long-survival group (*p* = 0.0068). (**c**), (**f**), (**i**), (**l**) In the HLA-A*2402-unmatched group, there was no difference in the percentages of PD-1+ and Tim-3+ CD4+ or CD8+ T cells between the patients with a long survival (*n* = 6) and the patients with a short survival (*n* = 15)

We classified the patients into two groups: a long-survival group and a short-survival group. We evaluated the PBMCs after the 3^rd^ cycle to evaluate the expression of PD-1 and Tim-3 on CD4+ and CD8+ T cells in 25 patients of the HLA-A*2402-matched group and 21 patients of the HLA-A*2402-unmatched group (Fig. 1). In the HLA-A*2402-matched group, the percentages of PD-1+ CD4+ and Tim-3+ CD4+ T cells in the patients of the short-survival group (*n* = 19; 3.1% ± 0.4% and 4.4% ± 0.5%, respectively) were significantly higher than in the patients of the long-survival group (*n* = 6; 1.4% ± 0.7% and 2.3% ± 0.4%, respectively; *p* = 0.0330 and *p* = 0.0282, respectively; Fig. 3b and 3e).

Similarly, in the HLA-A*2402-matched group, the percentages of PD-1+ CD8+ and Tim-3+ CD8+ T cells in the patients of the short-survival group (7.4% ± 1.7% and 7.6% ± 1.1%, respectively) were also significantly higher than in the patients of the long-survival group (2.0% ± 0.6% and 2.7% ±0.6%, respectively; *p* = 0.0046 and *p* = 0.0068, respectively; Fig. 3h and 3k).

In contrast, there was no significant difference for these factors in the HLA-A*2402-unmatched group (Fig. 3c, 3f, 3i, and 3l).

### Correlation between PD-1 and Tim-3 expression on CD4+ and CD8+ T cells in the patients with HLA-A*2402-matched group

We assessed the correlation between PD-1 and Tim-3 expression on CD4+ and CD8+ T cells in the patients with HLA-A*2402-matched group after the treatment. There was no correlation between PD-1 and Tim-3 expression on CD4 T cells (*r* = 0.3015, *p* = 0.1430) (Additional file 3: Figure S2a). However, PD-1 expression on CD8 T cells was significantly correlated with Tim-3 expression on CD8 T cells (*r* = 0.5385, *p* = 0.0055) (Additional file 3: Figure S2b).

### Changes in the CD4+ CD45RA- CD25high cells (Tregs) and CD11b + CD33+ cells (MDSCs)

We classified the patients into two groups: a long-survival group and a short-survival group. We assessed negative immune factors, focusing on Tregs and MDSCs, in the 46 patients of this study before and after the 3rd cycle treatment (Fig. 1). There was no significant difference before and after treatment (Additional file 4: Figure S3a and S3d). Next, we evaluated the prognostic differences between these factors according to the HLA-A*2402-matched group and the HLA-A*2402-unmatched group. Before and after treatment, there was no significant difference in the percentages of Tregs and MDSCs between the patients of the long-survival group and the patients of the short-survival group in the HLA-A*2402-matched group and the HLA-A*2402-unmatched group (Additional file 4: Figure S3b, S3c, S3e, and S3f).

## Discussion

Due to the very rapid and impressive progress in the area of cancer immunology [32], a large number of novel vaccine approaches for the treatment of cancer are being developed [33, 34]. However, useful biomarkers that can predict a better clinical outcome from immunotherapy have not yet been identified [15]. In this study, we investigated novel predictive biomarkers for immunotherapy by comparing the prognosis of 63 patients. We used HLA-A*2402-restricted peptides in this study. As such, the 36 patients in the HLA-A*2402-matched group are considered to comprise an immunological treatment group, while the 27 patients in the HLA-A*2402-unmatched group are considered to comprise a control group. The results of this study are useful because we could demonstrate the potential effectiveness of a peptide vaccine according to some biomarkers that could predict responsiveness to the vaccine treatment.

Firstly, a high expression level of PD-1 on CD4+ T cells might be the most useful predictor of poor OS, as seen by multivariate analysis with the Cox regression model (*p* = 0.0231; Table 2), and the log-rank test also showed that patients with a high expression level of PD-1 on CD4+ T cells had poorer OS than those with a low expression level of PD-1 on CD4+ T cells (*p* = 0.0036; Fig. 2a). However, in the HLA-A*2402-unmatched group, there was no difference between the patients with a high or low expression level of PD-1 on CD4+ T cells (*p* = 0.1191; Fig. 2b). These results support our hypothesis that a high expression level of PD-1 on CD4+ T cells could be used as a biomarker for response to immunotherapy.

PD-1 is a key immune checkpoint receptor that is expressed on activated T cells. PD-1 ligand 1 (PD-L1) is expressed on tumor cells in various cancers, and this expression on tumors is thought to contribute to tumor immune evasion [35]. Tim-3 is also an inhibitory receptor that is expressed on type 1 helper T cells and CTLs [36]. T cell exhaustion is a state of T cell dysfunction in the tumor microenvironment. It has been reported that the expression of PD-1 and Tim-3 on exhausted T cells results in reduced proliferation and effector functions in tumors [37]. The patients with a high expression level of PD-1 on CD4+ T cells might be unable to maintain the response of adaptive immune cells against cancer by vaccination. In this study, the induction of CTLs was also reduced in those with a high expression level of PD-1 on CD4+ T cells (Table 3). These results indicate that restoration of the insufficient antitumor immune response in patients with a high expression level of PD-1 on CD4+ T cells may be a viable approach for further improving the clinical efficacy of cancer immunotherapy.

After treatment, we found that the upregulation of PD-1 and Tim-3 expression on CD4+ T cells was significantly associated with a poor prognosis in the HLA-A*2402-matched group (*p* = 0.0330 and *p* = 0.0282, respectively; Fig. 3b and 3e). However, among the 21 patients of the HLA-A*2402-unmatched group, there was no significant difference between the patients with a high or low expression level of PD-1 or Tim-3 on CD4+ T cells. Similar to these results of PD-1 and Tim-3 expression on CD4+ T cells, after treatment, we found that the upregulation of PD-1 and Tim-3 expression on CD8+ T cells was significantly associated with a poor prognosis in only the HLA-A*2402-matched group (*p* = 0.0046 and *p* = 0.0068, respectively; Fig. 3h and 3k). These results also confirmed our hypothesis that the expression of PD-1 and Tim-3 on CD4+ and CD8+ T cells could be used as a biomarker for immunotherapy outcome. Vaccine therapy is designed to attack cancers by stimulating T cells and directing them to recognize and act as TAA-specific T cells. In our study, the expression of PD-1 and Tim-3 on both CD4+ and CD8+ T cells was significantly upregulated in the short-survival group after treatment. It is extremely difficult for vaccine therapy to enhance immune responses in the immunosuppressive state, which may account for why there might be no statistical difference between the HLA-A*2402-matched group and the HLA-A*2402-unmatched group. Blockade of PD-1 and PD-L1 interactions can reverse T cell exhaustion and restore antigen-specific T cell responses [38]. These results indicate that the combination of vaccine therapy with an immune checkpoint blockade might be effective in advanced PC patients.

It has been reported that TIM-3 is co-expressed with PD-1 on exhausted T cells [39, 40]. Our present study also showed that the expressions of PD-1 and TIM-3 on CD8 T cells were both significantly upregulated and had a significant positive correlation after treatment (Fig. 3h, 3k and Additional file 3: FigureS2b). In this study, although the upregulation of PD-1 expression on CD4+ cells was significantly associated with a poor clinical outcome before treatment, there was no significant difference of Tim-3 (Table 2). These results may indicate that TIM-3 could be expressed exclusively on T cells that co-express PD-1, whereas, PD-1 expression might not be required for Tim-3 co-expression.

It has been reported that high CTL responses to multiple peptides after vaccination are a possible biomarker for a long survival in vaccinated patients [23, 29, 31]. In this study, we observed that a low NLR and low number of Tregs were also significantly associated with a high CTL response (Table 3). Hence, we speculated that a low NLR and number of Tregs may be related to predictive biomarkers. The NLR is an easily calculated and simple marker of the systemic inflammatory response [41]. Several studies have suggested that a high NLR is associated with a poor prognosis in patients with various cancers [18, 42, 43]. A decreased number of lymphocytes diminishes the antitumor immune response and worsens the prognosis [44]. However, the NLR was not significantly correlated with a poor prognosis in this study, although the small sample size may have accounted for this.

Tregs are considered to be one of the most powerful inhibitors of antitumor immunity and is correlated with a poor prognosis [45]. GEM has the potential to enhance the antitumor effects of cancer immunotherapy by suppressing the induction of Tregs and MDSCs [27, 46]. Although we administered combination therapy with vaccine and GEM in the present study, we did not find any significant decrease in these cell populations. These results indicate that Tregs are not associated with a poor prognosis. Therefore, the combination of immunotherapy and another chemotherapy that inhibits these immunosuppressive cells might be attractive for advanced PC patients.

## Conclusions

In conclusion, although the number of patients in this study was very limited, a high expression level of PD-1 on CD4+ T cells may be a very promising biomarker for predicting the prognosis of PC patients with vaccination. The expression of PD-1 and Tim-3 on CD4+ and CD8+ T cells may also be a useful biomarker for predicting the efficacy of cancer immunotherapy. Our results indicate that the upregulation of PD-1 and Tim-3 expression on CD4+ and CD8+ T cells may restrict T cell responses in advanced PC patients. As such, combination immunotherapy with blockade of PD-1 and Tim-3 that restores T cell responses may be a potential therapeutic approach for treating advanced PC patients.

## Additional files

Additional file 1: Figure S1. Positivity for antigen-specific T cell responses was quantitatively defined according to the evaluation tree algorithm. In brief, the peptide-specific spots (SS) were the averages of triplicates calculated by subtracting the HIV peptide-pulsed stimulator well from the immunized peptide-pulsed stimulator well. The %SS means the percentage of SS among the average spots of the immunized peptide-pulsed stimulator well. The antigen-specific T cell responses were classified into four grades (−, +, ++, and +++) depending on the number of peptide-specific spots and the invariability of the peptide-specific spots at different responder/stimulator ratios. SS, peptide-specific spots; R1, responder/stimulator ratio = 1; R2, responder/stimulator ratio = 0.5; R3, responder/stimulator ratio = 0.25; R4, responder/stimulator ratio = 0.125. (TIF 332 kb)
Additional file 2: Table S1. Comparison of prognostic factors according to the numbers of peptide-specific responses (*n* = 36 HLA-A*2402-matched patients). (DOCX 28 kb)
Additional file 3: Figure S2. Correlation between PD-1 and Tim-3 expression on CD4+ and CD8+ T cells in the patients with HLA-A*2402-matched group after 3rd cycle treatment. (a) There was no correlation between PD-1 and Tim-3 expression on CD4 T cells (*r* = 0.3015, *p* = 0.1430). (b) PD-1 expression on CD8 T cells was significantly correlated with Tim-3 expression on CD8 T cells (*r* = 0.5385, *p* = 0.0055). (TIF 35 kb)
Additional file 4: Figure S3. Frequency of CD4+ CD45RA- CD25high cells and CD11b + CD33+ cells. (a), (c) There were no differences in the percentages of CD4+ CD45RA- CD25high cells and CD11b + CD33+ cells in the 46 patients before and after treatment. (b), (e) Before and after treatment, there were no differences in the percentages of CD4+ CD45RA- CD25high cells and CD11b + CD33+ cells between the patients with a long survival (*n* = 6) and the patients with a short survival (*n* = 19) in the HLA-A*2402-matched group. (c), (f) Before and after treatment, there were no differences in the percentages of CD4+ CD45RA- CD25high cells and CD11b + CD33+ cells between the patients with a long survival (*n* = 6) and the patients with a short survival (*n* = 15) in the HLA-A*2402-unmatched group. (TIF 60 kb)

## Abbreviations

CA19-9: Carbohydrate antigen 19-9
CEA: Carcinoembryonic antigen
CI: Confidence interval
CRP: C-reactive protein
CTLs: Cytotoxic T lymphocytes
GEM: Gemcitabine
Hb: Hemoglobin
HLA: Human leukocyte antigen
HR: Hazard ratio
IL-6: Interleukin-6
MDSCs: Myeloid-derived suppressor cells
MST: Median survival time
NLR: Neutrophil-lymphocyte ratio
OS: Overall survival
PBMCs: Peripheral blood mononuclear cells
PC: Pancreatic cancer
PD-1: Programmed death-1
PD-L1: PD-1 ligand 1
TAA: Tumor-associated antigens
Tim-3: T cell immunoglobulin mucin-3
Tregs: Regulatory T cells
VEGFs: Vascular endothelial growth factor receptors
