# Supplementary figures and images for: Predictive biomarkers for the efficacy of peptide vaccine treatment: based on the results of a phase II study on advanced pancreatic cancer
Source: J Exp Clin Cancer Res. 2017 Feb 28;36:36. doi: 10.1186/s13046-017-0509-1 (PMC5329922; doi:10.1186/s13046-017-0509-1)

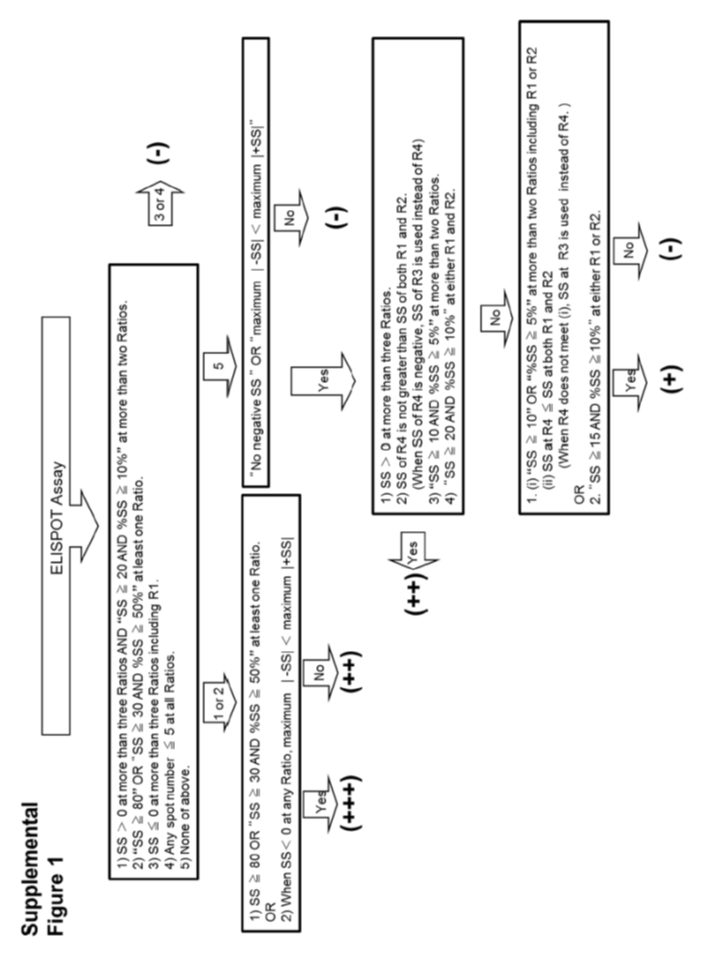

Supplement: Additional file 1: Figure S1. — Positivity for antigen-specific T cell responses was quantitatively defined according to the evaluation tree algorithm. In brief, the peptide-specific spots (SS) were the averages of triplicates calculated by subtracting the HIV peptide-pulsed stimulator well from the immunized peptide-pulsed stimulator well. The %SS means the percentage of SS among the average spots of the immunized peptide-pulsed stimulator well. The antigen-specific T cell responses were classified into four grades (−, +, ++, and +++) depending on the number of peptide-specific spots and the invariability of the peptide-specific spots at different responder/stimulator ratios. SS, peptide-specific spots; R1, responder/stimulator ratio = 1; R2, responder/stimulator ratio = 0.5; R3, responder/stimulator ratio = 0.25; R4, responder/stimulator ratio = 0.125. (TIF 332 kb) [file 13046_2017_509_MOESM1_ESM.tif]

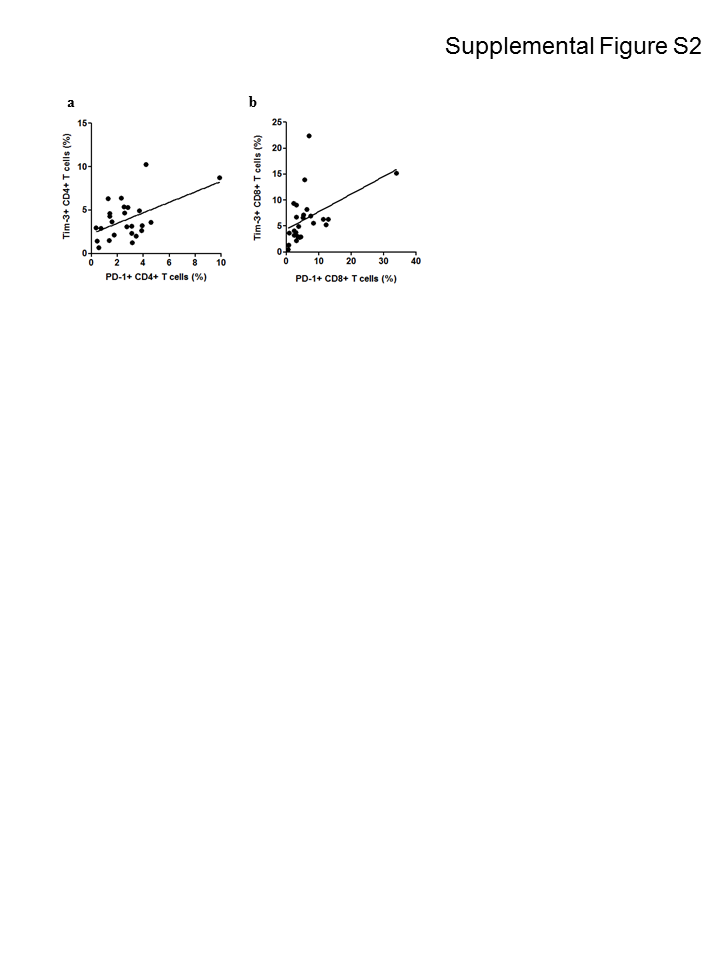

Supplement: Additional file 3: Figure S2. — Correlation between PD-1 and Tim-3 expression on CD4+ and CD8+ T cells in the patients with HLA-A*2402-matched group after 3rd cycle treatment. (a) There was no correlation between PD-1 and Tim-3 expression on CD4 T cells (r = 0.3015, p = 0.1430). (b) PD-1 expression on CD8 T cells was significantly correlated with Tim-3 expression on CD8 T cells (r = 0.5385, p = 0.0055). (TIF 35 kb) [file 13046_2017_509_MOESM3_ESM.tif]

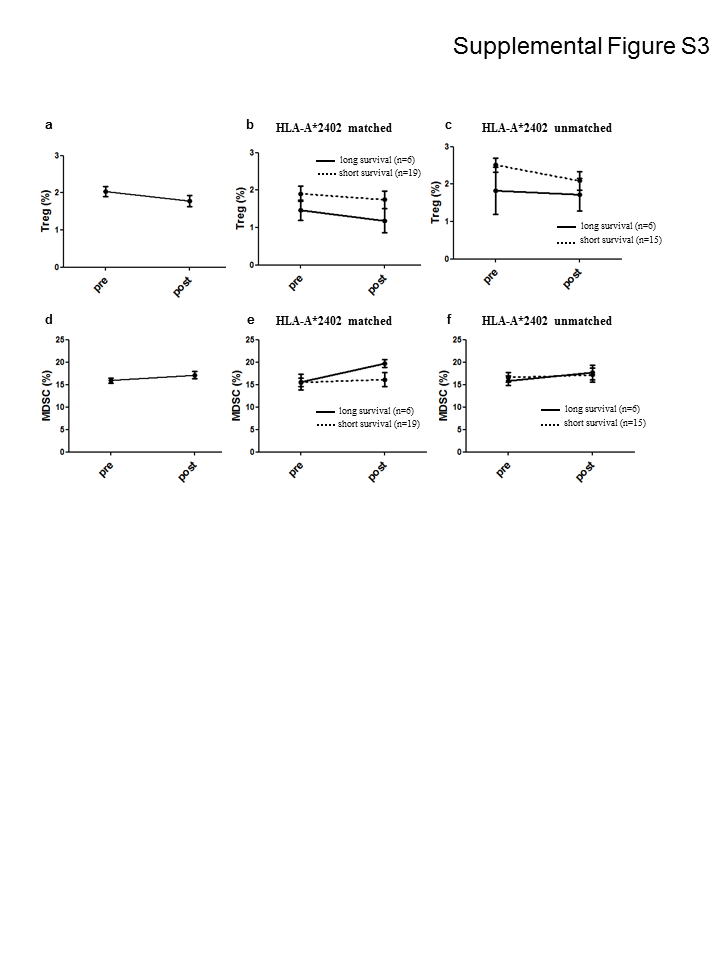

Supplement: Additional file 4: Figure S3. — Frequency of CD4+ CD45RA- CD25high cells and CD11b + CD33+ cells. (a), (c) There were no differences in the percentages of CD4+ CD45RA- CD25high cells and CD11b + CD33+ cells in the 46 patients before and after treatment. (b), (e) Before and after treatment, there were no differences in the percentages of CD4+ CD45RA- CD25high cells and CD11b + CD33+ cells between the patients with a long survival (n = 6) and the patients with a short survival (n = 19) in the HLA-A*2402-matched group. (c), (f) Before and after treatment, there were no differences in the percentages of CD4+ CD45RA- CD25high cells and CD11b + CD33+ cells between the patients with a long survival (n = 6) and the patients with a short survival (n = 15) in the HLA-A*2402-unmatched group. (TIF 60 kb) [file 13046_2017_509_MOESM4_ESM.tif]
