# Supplementary material for: Predictive biomarkers for the efficacy of peptide vaccine treatment: based on the results of a phase II study on advanced pancreatic cancer
Source: J Exp Clin Cancer Res. 2017 Feb 28;36:36. doi: 10.1186/s13046-017-0509-1 (PMC5329922; doi:10.1186/s13046-017-0509-1)
Supplement: Additional file 2: Table S1. — Comparison of prognostic factors according to the numbers of peptide-specific responses (n = 36 HLA-A*2402-matched patients). (DOCX 28 kb) [file 13046_2017_509_MOESM2_ESM.docx]

| Table S1. Comparison of prognostic factors according to the numbers of peptide-specific responses (n = 36 HLA-A*2402-matched patients) | | | |
| --- | --- | --- | --- |
| Variables | the number of peptide specific response | | p-value |
|  | 0 | 1, 2 or 3 |  |
| the number of patients | 5 | 31 |  |
| Age | 68.6 + 6.3 | 62.0 + 2.2 | 0.2715 |
| Gender |  |  | 0.1672 |
| Male | 4 | 13 |  |
| Female | 1 | 18 |  |
| Stage (UICC) |  |  | 0.6033 |
| III | 0 | 7 |  |
| IV | 5 | 21 |  |
| Recurrence | 0 | 3 |  |
| NLR | 3.91 + 0.49 | 2.33 + 0.17 | **0.0153** |
| CRP | 1.89 + 0.63 | 0.63 + 0.18 | **0.0153** |
| IL-6 | 2.73 + 1.30 | 16.51 + 14.53 | 0.8191 |
| PD-1+ CD4+ T cell | 2.96 + 0.91 | 1.97 + 0.24 | 0.1433 |
| Tim-3+ CD4+ T cell | 3.40 + 0.68 | 3.63 + 0.58 | 0.7488 |
| PD-1+ CD8+ T cell | 4.72 + 0.72 | 4.45 + 0.45 | 0.7144 |
| Tim-3+ CD8+ T cell | 3.79 + 0.71 | 5.12 + 0.49 | 0.2928 |
| Treg | 2.51 + 0.44 | 1.74 + 0.13 | 0.0639 |
| MDSC | 17.35 + 2.71 | 14.78 + 0.77 | 0.2002 |
| Statistical significant results are highlighted in bold letters. | | |  |
| Abbreviations: HLA, human leukocyte antigen; CI, confidence interval; UICC, Union for International Cancer; NLR, neutrophil lymphocyte ration; CRP, C-reactive protein; IL-6, interleukin-6; PD-1, Programmed death-1; Tim-3, T cell immunoglobulin mucin-3; Treg, Regulatory T cell; MDSC, Myeloid-derived suppressor cell. | | | |
